# New Insights into Detection of a Dendrobine Compound From a Novel Endophytic *Trichoderma longibrachiatum* Strain and Its Toxicity Against Phytopathogenic Bacteria

**DOI:** 10.3389/fmicb.2020.00337

**Published:** 2020-03-12

**Authors:** Surendra Sarsaiya, Archana Jain, Xiaokuan Fan, Qi Jia, Quan Xu, Fuxing Shu, Qinian Zhou, Jingshan Shi, Jishuang Chen

**Affiliations:** ^1^Key Laboratory of Basic Pharmacology of Ministry of Education, Zunyi Medical University, Zunyi, China; ^2^Joint International Research Laboratory of Ethnomedicine of Ministry of Education, Zunyi Medical University, Zunyi, China; ^3^Bioresource Institute for Healthy Utilization, Zunyi Medical University, Zunyi, China; ^4^College of Biotechnology and Pharmaceutical Engineering, Nanjing Tech University, Nanjing, China

**Keywords:** chromatography, dendrobine, *Dendrobium nobile*, endophytic fungi, phytopathogenic bacteria, *Trichoderma longibrachiatum*

## Abstract

*Dendrobium nobile* is the only plant that could produce the natural bioactive dendrobine. No other source of dendrobine has been found to date except from *D. nobile* and via chemical synthesis. In this study, we aimed to examine the potential fungal endophyte isolated from *D. nobile* stem segments using the molecular method and to detect dendrobine compound through high-performance liquid chromatography (HPLC), gas chromatography–mass spectrometry (GC-MS), and liquid chromatography with tandem mass spectrometry (LC-MS/MS) and their metabolite for their antibacterial activity. The potential dendrobine producer strain was recognized as *Trichoderma longibrachiatum* based on molecular DNA sequencing and GenBank databases. The *T. longibrachiatum* MD33 produced dendrobine and other compounds in a potato dextrose medium (PDM), as confirmed by HPLC retention time peak analysis. The HPLC results revealed that *T. longibrachiatum* MD33 biomass showed a peak retention time of 5.28 ± 0.2 min, similar to wild *D. nobile* stem dendrobine (5.32 ± 0.2 min) and standard chemical reference dendrobine (5.30 ± 0.2 min), indicating the presence of dendrobine in the fungal biomass. Results of GC-MS and LC-MS analysis revealed that *T. longibrachiatum* MD33 produced the same molecular weight (263 in GC-MS and 264.195 in LC-MS) of dendrobine as compared with standard chemical reference dendrobine and *D. nobile* dendrobine. Antibacterial activity data revealed that *T. longibrachiatum* MD33 produced the strongest bactericidal activity against *Bacillus subtilis*, *Bacillus mycoides*, and *Staphylococcus* species, and the diameter of the bacterial growth inhibition zone was 12 ± 0.2, 9 ± 0.2, and 8 ± 0.2 mm, respectively. To the best of our knowledge, this was the first study to investigate *T. longibrachiatum* as a dendrobine producer, and the results revealed that *T. longibrachiatum* was directly involved in the potential production of a similar bioactive compound to *D. nobile* (dendrobine). In addition, the *T. longibrachiatum* metabolite exhibited potent antibacterial activity and can be a potential strain for medical and industrial purposes.

## Introduction

Plant tissues are intracellularly colonized by a complex group of endophytic microbial species, where they play a vital role in plant development, health, and protection. These endophytic microbes, especially fungi, have the ability to promote plant development via indirect and direct mechanisms. Endophytic fungi can directly help host plants through the production of several plant development regulators and by enabling nutrient uptake. In addition, they can keep the host plant healthy through indirect responses by producing substances such as antibiotics and siderophores to inhibit phytopathogens (Bahroun et al., 2018). Recently, the medicinal plant-based wastewater remediation approaches are highlighted due to their being comparatively cheap and ecologically advantageous, compared to other common technological approaches. There are several medicinal plants species known for their phytoremediative abilities (Hegazy et al., 2011; Santal et al., 2019). The *Dendrobium* phytoremediation potentials of plant species have been considered in many previous researches (Sarawaneeyaruk et al., 2014; Sutantoa et al., 2016; Santal et al., 2019).

Owing to health hazards and harmful effects associated with the indiscriminate use of artificial drugs and antibiotics, there has been growing attention to the practice of biomedicine. In the past 20 years, more than half of the market drugs were developed from bioresources (Pham et al., 2019). *Dendrobium* plants belonging to the Orchidaceae family have been attributed as earliest sources of medicine over extended periods in Asia, Australia, and Europe. More than 1100 species were testified worldwide and have been verified to be an upright source of bioactive metabolites (Khamchatra et al., 2016). The orchid family, Orchidaceae, represents earlier branches of the evolutionary tree and is possibly 120 million years old. It is one of the earlier families of angiosperms in which majority of plants are composed of highly evolved flowering plants, with approximately 25,000 to 35,000 species belonging to 750 to 900 genera (Lam et al., 2015; Sarsaiya et al., 2019b, 2020).

*Dendrobium*, referred to as Shihu in traditional Chinese medicine, is widely distributed throughout China and includes 78 species. Approximately 30 species (of 78 species) have been used as chief medicinal agents and food, such as tea or soup ingredients, for many centuries (Deng et al., 2018). The compounds found in *Dendrobium*, including dendrobine and related compounds, can boost human immunity besides preventing and improving metastatic cancer and have shown promising therapeutic effects on Alzheimer’s disease (Nie et al., 2016). This medicinal plant has been used to prevent or treat illnesses owing to the presence of bioactive compounds in their cells and have been added to drugs since ancient times (Elgorban et al., 2019). However, dendrobine is not produced in large quantities by the *D. nobile* plant species because of its slow growth. The quantity of *D. nobile* dendrobine is low to fulfill the current industrial and research requirements (Jiang et al., 2018; Zhang et al., 2018).

Endophytic fungus has also been described to produce active enzymes and metabolites, which may be used to produce pharmaceutical products. Endophytic fungus can form a consortium of unstable carbon-based complexes (aldehydes, ketones, hydrocarbons, alcohols, heterocycles, thioesters, thioalcohols, phenols, and their byproducts) through substantial antimicrobial actions counter to plants and human pathogens (Manganyi et al., 2018; Jain et al., 2019). Most researchers are investigating the diversity and functional characteristics of fungal endophytes as biocontrol. They have focused on economically imperative crops or indigenous herbal class. The dynamic relationship between the host and its fungal endophytes has not been fully understood to date. However, a few endophytic fungi are recognized and categorized based on their molecular characteristics (Singh et al., 2017; Hoysted et al., 2018). To the best of our knowledge, this is the first study in China to analyze endophytic fungal diversity in *Dendrobium nobile* and to identify fungal metabolites, such as dendrobine and similar compounds, for the development of pharmacologically and industrially imperative bioactive compounds.

Some fungal endophytes are known to produce bioactive metabolites, and these metabolites may be similar to those produced by the host. For example, *Taxomyces andreanae*, a paclitaxel-producing fungal endophyte, was effectively recovered from the yew tree *Taxus brevifolia*. A study using a *Chaetomium* sp. reported the possible industrial production of paclitaxel (1124.34 μg/L) under optimized fermentation conditions. A gibberellin-producing *Penicillium commune* strain has been recovered from the *Sesamum indicum* plant (Elgorban et al., 2019). Many fungal endophytes have the potential to biosynthesize numerous bioactive natural compounds that may indirectly or directly be applied as therapeutic mediators in the treatment of various diseases. Fungal endophytes that produce the host herbal secondary metabolites with beneficial value or potential have been discovered (Dey et al., 2017). The detection of bioactive metabolites produced by fungal endophytes by using compounds simulated to resemble associated host herbal metabolites is important for both industrial and educational purposes (Kusari et al., 2012; Yu et al., 2017). In contrast, fewer efforts have been made to explore bioactive compounds similar to plant compounds produced by fungal endophytes (Raghavendra et al., 2017). Bacterial contaminations constitute a major plant health problem due to wastewater in developing and developed countries, which directly or indirectly influence growth. In recent years, the change of resistance of bacterial pathogens against natural compounds has become a problematic issue caused by the unselective use of natural compounds. Natural compounds have been called miracle natural active substances, but for more than 60 years of use, the efficacy of current natural active substances has been reduced due to the continuing emergence of natural active substance-resistant bacterial population in wastewater and the adaptations by bacterial pathogens to commonly used antibacterial compounds (Donga et al., 2017). Due to such issues, it is important to explore novel natural compounds, which have the ability to show effective antibacterial activity.

The present study was therefore performed to determine the presence of *Trichoderma longibrachiatum* endophytic fungal strain in *D. nobile* plants from Chishui, Guizhou, China. In addition, we aimed to assess the potential of the fungi to produce dendrobine compounds through HPLC, gas chromatography–mass spectrometry (GC-MS), and liquid chromatography with tandem mass spectrometry (LC-MS/MS) analysis and to determine the antibacterial effects of its metabolite against phytopathogenic bacteria.

## Materials and Methods

### Processing of Samples for the Isolation of Endophytic Fungi

**FIGURE 1:**
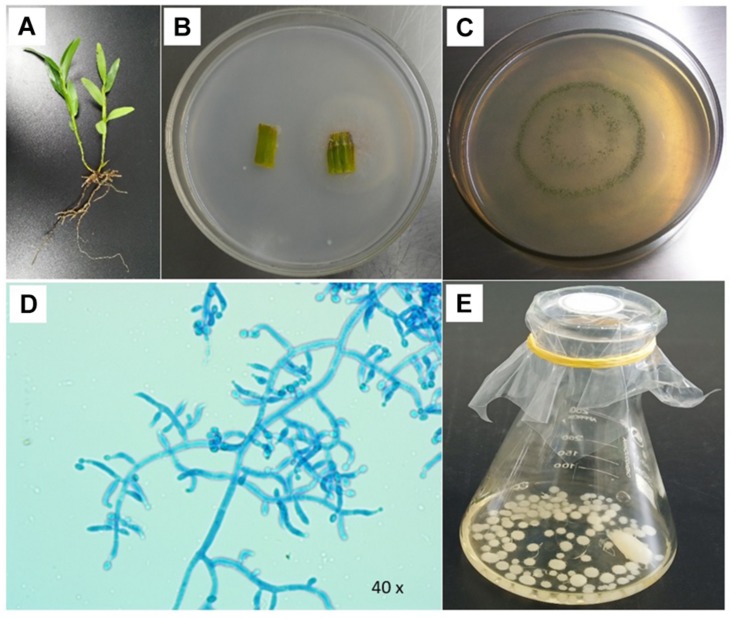
Examination of *Trichoderma longibrachiatum* MD33 isolated from wild *Dendrobium nobile* stem segment. **(A)** Wild *D. nobile* plant. **(B)** Endophytic fungal culture emerge from stem. **(C)** Pure culture of *T. longibrachiatum* MD33. **(D)** Microscopic characteristics of *T. longibrachiatum* MD33. **(E)** *T. longibrachiatum* MD33 mycelial growth in the potato dextrose liquid medium.

The endophytic fungi were isolated from the stem segments of single wild *D. nobile*, which was collected from the native agricultural farm area of Jinshishi, Chishui, China, and processed in the Bioresource Institute for Health Utilization, Zunyi Medical University, Zunyi, China. The processing of samples was performed according to the previous process of Sarsaiya et al. (2019a) and Sarsaiya et al. (2020). The stem samples of wild *D. nobile* were considered for processing and isolation. The samples (1.5 cm per piece) were inoculated onto PDA media plates. The plates were placed in an incubator at 25°C for 5 days. After 5-day incubation, the fungal hyphal growths emerged from the plant samples (Figure 1). These were recovered and purified by sub-culturing from growing hyphal tips.

### Identification of Plant Endophytic Fungi

The potential dendrobine producing endophytic fungal genus was identified by using lactophenol cotton blue staining and microscopic observations (Ellis, 1976; Barnett and Hunter, 1998). For molecular identification, the DNA was extracted and polymerase chain reaction (PCR) was performed to amplify the internal transcribed spacer (ITS) regions for endophytes using a standard protocol (Manganyi et al., 2018).

### Sequence Analysis of PCR Products

The amplified ITS regions of PCR samples were sequenced by Tsingke, Beijing, China. Sequence data were analyzed by using the BLAST (basic local alignment search tool) Web Tool on the NCBI (National Center for Biological Information)^1^ to validate the similarity of species of the amplified sequences as compared with NCBI reference sequence. MEGA (molecular evolutionary genetics analysis) 7.0 software was used for phylogeny tree study.

### Screening Medium for the Detection of Dendrobine Compounds

Potato dextrose medium was used for the synthesis of dendrobine compounds from the isolated endophytic fungi. Erlenmeyer flasks (250 ml) contained 100 ml of PDM in triplicate (initial pH 6.4). Two disks of 5-day-old fungal culture were inoculated separately on PDM and incubated at 25 ± 2°C for 14 days. The fungal culture biomass and metabolites were tested for the presence of dendrobine compounds through HPLC. The culture biomass and metabolite have shown dendrobine peak, which was further analyzed by using the GC-MS and LC-MS/MS analysis. Sample processing for the dendrobine detection through HPLC, GC-MS, and LC-MS/MS analysis is as follows:

### HPLC Analysis for the Detection of Dendrobine Compounds

#### Preparation of Standard Dendrobine

Oven-dried powder of *D. nobile* stem was used for the extraction of dendrobine alkaloids. The powder was mixed with absolute ethanol and boiled at 90°C for 2 h. After extraction, the contents were filtered through Whatman filter paper, and the filtrate was transferred to sample tubes for HPLC analysis by comparing with the peak retention time of standard chemical reference dendrobine (purchased from Chengdu DeSiTe Biological Technology Co., Ltd., Chengdu, China, with purity greater than 99%).

#### Separation of Fungal Biomass and Metabolite

The endophytic fungal biomass and metabolite were separated from the Erlenmeyer flask by using the Water-Circulation Multifunction Vacuum Pump (Zhengzhou Greatwall Scientific Industrial and Trade Co., Ltd, Henan, China).

#### Freeze Drying of Fungal Biomass Samples

The test endophytic fungal biomass, after the separation through a Water Circulation Multifunction Vacuum Pump, was freeze dried at −40°C at 669 mbar for 10 h by using a ModulyoD-230 Freeze Dryer (Thermo Electron Corporation, Milford, MA, United States).

#### HPLC Analysis

The freeze-dried biomass (100 mg power of biomass used) and metabolite samples were mixed separately with 600 μl of acetonitrile in a 1.5-ml centrifuge tube. The tubes were vortex mixed for 1 min and centrifuged at 15,000 rpm (20,627 × *g*) for 15 min. Dendrobine and related compounds were analyzed using HPLC Agilent 1100 (United States of America) equipped with a C18 column (150 × 4.6 mm, 5 μm) maintained at 35°C. The mobile phase comprised 0.1% formic acid and acetonitrile with gradient elution at a flow rate of 1 ml/min. The injection volume was 20 μl. The ultraviolet (UV) wavelength was 205 nm. The retention peak time of dendrobine and its related compounds was calculated for all the trials through chromatograms.

### Detection of Dendrobine by GC-MS

The following conditions were maintained in the GC-MS for the detection of dendrobine: Instrument: Agilent Technologies 6890N Network GC system; 5973 Network Mass selective detector; 7683B series Injector; Column: Capillary column Agilent 190915-433 HP-5MS (30.0 M × 250 μm × 0.25 μm); Initial temperature 150°C, keep 5 min, 10°C min^–1^, Temperature-programed 250°C, keep 5 min; Inlet temperature of 250°C, detector temperature of 250°C; carrier: N_2_, Flow rate: 1 ml min^–1^, sample volume 1 μl.

### Detection of Dendrobine by LC-MS/MS

#### Intracellular Dendrobine Extraction From Fungal Biomass

For the extraction of dendrobine from fungal biomass, the 100 mg of dry biomass was powdered in a mortar pestle, soaked with 50 ml of chloroform into the 50-ml centrifuge tube for 12 h at 180 rpm. From the mixture, the 40-ml liquid part was extracted by using the 5-ml Eppendorf micropipette. The plant dendrobine was also processed the same as the intracellular dendrobine extraction process. The chloroform phase was separated by evaporation at 35°C under a rotary evaporator (Rotary Evaporator N-1300V-W, EYELA, United States). After evaporation, the remaining residue was re-dissolved in 5 ml of chloroform and filtered through a 0.22-mm filter prior to analysis.

#### *D. nobile* Dendrobine Extraction

For the extraction of dendrobine from *D. nobile*, the freeze-dried stem sample (100 mg) of *D. nobile* was powdered after freeze drying (ModulyoD-230 Freeze Dryer; Thermo Electron Corporation, Milford, MA, United States) in a mortar pestle, soaked with 50 ml of chloroform for 12 h at 180 rpm. From the mixture, the 40-ml liquid part was separated. The chloroform phase was separated from the aqueous phase and evaporated at 35°C under a rotary evaporator. The remaining residue was re-dissolved in 5 ml of chloroform and filtered through a 0.22-mm filter prior to analysis.

#### Standard Dendrobine Solution Preparation

The stock solution of dendrobine (20.0 μg/ml) was prepared in chloroform. The 20, 80 ng/ml, and 20.0 μg/ml working standard solutions of the dendrobine were prepared from the dendrobine stock solution by dilution with chloroform. The dendrobine standard was purchased from Chengdu DeSiTe Biological Technology Co., Ltd., Chengdu, China, with a purity greater than 99%.

#### Detection of Dendrobine by LC-MS/MS

LC-MS is used for non-volatile and thermally fragile molecules. The detection of dendrobine was performed via the UHPLC system (Thermo Scientific DionexUltiMate 3000, Golden Valley, Minnesota, United States) with a column (150 × 2.1 mm) and a mobile phase consisting of 0.1% formic acid:acetonitrile at 95:5 (v/v) with a flow rate of 0.3 ml/min, a sheath gas flow rate of 35 arbitrary units, an auxiliary gas flow rate of 15 arbitrary units, a spray voltage of 3.5 kV, a capillary temperature of 350°C, and an aux gas heater temperature of 400°C. Identification of dendrobine was accomplished by comparison of retention times, molecular weight (264.195), and LC-MS fragmentation patterns with authentic chemical reference standard (dendrobine standard was purchased from Chengdu DeSiTe Biological Technology Co., Ltd., Chengdu, China, with a purity greater than 99%) and *D. nobile* plant stem dendrobine.

### Toxicological Bioassay of Fungal Metabolite Against Plant Pathogenic Bacteria

Toxicological bioassay used in this research was performed as per the standard procedure (Bahroun et al., 2018). The pure pathogenic identified cultures of *Bacillus subtilis*, *Bacillus mycoides*, and *Staphylococcus* sp. strains were obtained from the Bioresource Institute for Health Utilization, Zunyi Medical University, Zunyi, China, for antibacterial study of fungal metabolite. A fresh culture of these selected pathogenic strains was transferred into 1 ml of sterile H_2_O, and a 100-μl portion was spread over solidified PDA plates. Five-millimeter-diameter filter paper disks immersed in fungal metabolite were placed at equal distances on the inoculated PDA plates. All bacterial isolations were done in triplicate and transferred to an incubator for 1 h incubation at 4°C to ensure diffusion of compounds from the fungal metabolite disks. Then, the test plates were incubated at 25°C for 24 h in a bacteriological incubator. The size of the bacterial growth inhibition zone was calculated in millimeters (mm). The standard formula (Balouiri et al., 2016) was applied to the inhibition zone size.

Inhibition zone = average diameter of the colony−5 mm (diameter)

### Statistical Analysis

All data were expressed as means ± standard deviation (S.D.). The comparison between different treatments was performed by a one-way ANOVA using SPSS version 20.0 software. A value of *p* < 0.05 was considered statistically significant.

## Results

### Endophytic Fungus Isolation and Identification

The *Trichoderma* was isolated and identified through ITS sequencing. It was confirmed from the NCBI web tool that the isolated fungi had maximum homology with *T. longibrachiatum*, and the investigated organism was confirmed to be *T. longibrachiatum*. The *Trichoderma* species was identified as *longibrachiatum* based on sequence identities of ≥100% with GenBank references, which belong to order Hypocreales and family Hypocreaceae. On the other hand, the *T. longibrachiatum* MD33 was identified at the species level based on sequence identities of ≥100% with its neighboring GenBank match. BLAST analysis showed that the isolated ITS sequence had 99% similarity with the ITS sequence of *T. longibrachiatum* (Accession no. NR_120298.1).

### Screening for the Detection of Dendrobine Compounds Through HPLC

**FIGURE 2:**
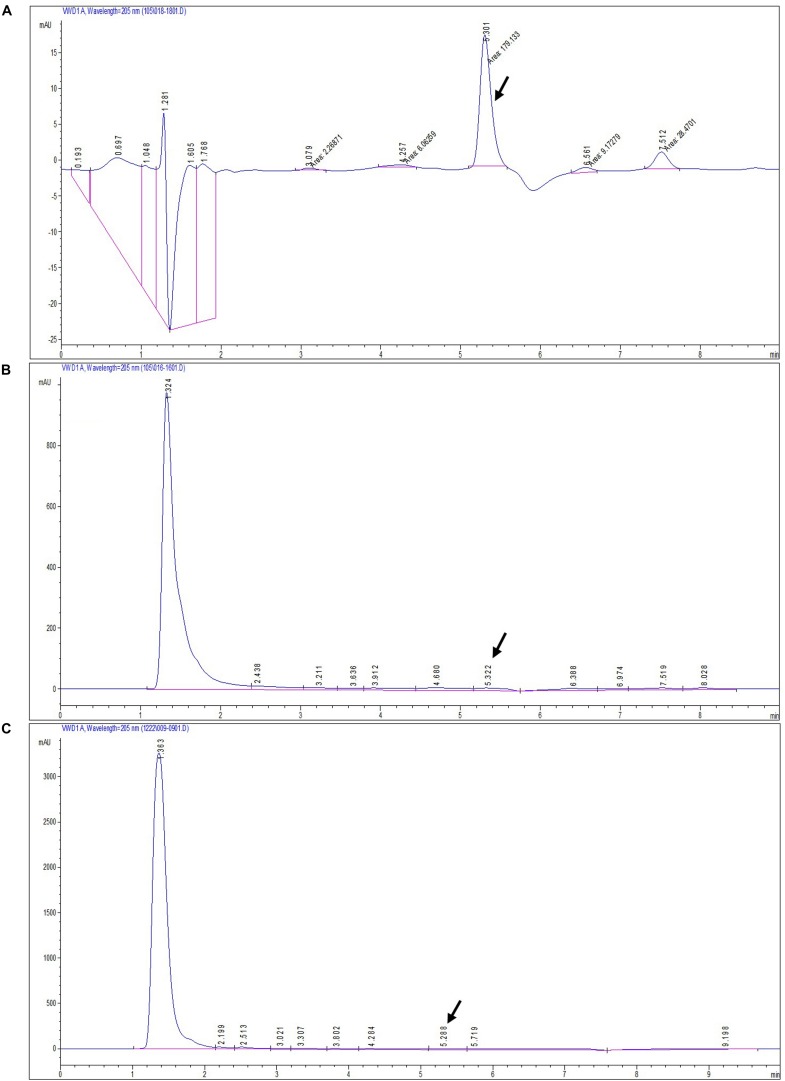
HPLC chromatograms of standard dendrobine **(A)**, *D. nobile* plant alkaloid **(B)**, and fungal dendrobine and other related compounds **(C)**. Dendrobine-specific peak was observed at 5.3 ± 0.02.

The secondary metabolite of the *T. longibrachiatum* MD33 strain was assessed by metabolite analysis through HPLC investigation to detect dendrobine compounds. The endophytic fungi were recovered from wild *D. nobile* stem segments and have shown great potential to release bioactive compounds through submerged fermentation and were, therefore, commonly used to produce natural active compounds. Based on the screening of all endophytic fungi for dendrobine and other related compounds, it was observed that *T. longibrachiatum* MD33 biomass samples showed the ability to produce compounds similar to dendrobine as compared with reference peaks and other related compounds. The fungal dry biomass of *T. longibrachiatum* was 1.36 ± 0.2 g after 14 days of incubation. Only *T. longibrachiatum* MD33 strain showed dendrobine peak as compared with standard chemical reference dendrobine and wild *D. nobile* stem dendrobine. HPLC analysis data of *T. longibrachiatum* MD33-fermented metabolites did not show any dendrobine peaks. The results revealed that the *T. longibrachiatum* MD33 biomass sample shared similar peak retention time to standard chemical reference dendrobine and *D. nobile* stem alkaloid (Figure 2 and Table 1). Dendrobine of *T. longibrachiatum* MD33 fungal endophyte and *D. nobile* stem alkaloid samples were identified based on peak retention time relative to that of the reference standard (dendrobine; >99% purity). The retention time of dendrobine was 5.28 ± 0.2 min and that of other compounds was 1.36 ± 0.2, 3.02 ± 0.2, and 4.28 ± 0.2 min. Many other peaks were also recorded at retention times of 3.02 ± 0.2, 4.28 ± 0.2, and 5.28 ± 0.2 min compared with the standard dendrobine, while dendrobine peak in both showed the same retention time (5.28 ± 0.2 min; Figure 2). Furthermore, the peak retention time of *D. nobile* stem alkaloid was similar to that of fungal metabolite (retention time 1.32 ± 0.2, 3.21 ± 0.2, and 5.32 ± 0.2 min). The retention time of 5.28 ± 0.2 min was similar in all HPLC chromatograms (Figure 2 and Table 1), indicating the presence of dendrobine compounds in the fungal metabolite.

**TABLE 1:** Comparison of HPLC, GC-MS, and LC-MS/MS analysis for the dendrobine peak retention times and its molecular weight.

Sample type	Dendrobine peak retention time (min)		
	
	HPLC	GC-MS	LC-MS/MS
	5.30 ± 0.02	14.97 ± 0.02	7.51 ± 0.02
*Trichoderma longibrachiatum* MD33	5.28 ± 0.02	14.96 ± 0.02	7.51 ± 0.02
Chemical reference standard dendrobine	5.30 ± 0.02	14.96 ± 0.02	7.57 ± 0.02
Wild *Dendrobium nobile* stem dendrobine	5.32 ± 0.02	14.97 ± 0.02	7.48 ± 0.02
Dendrobine molecular weight	NA	263	264.195

*All the values are the standard deviation of three replicates; NA: not available.*

### Detection of Dendrobine by GC-MS Analysis

**FIGURE 3:**
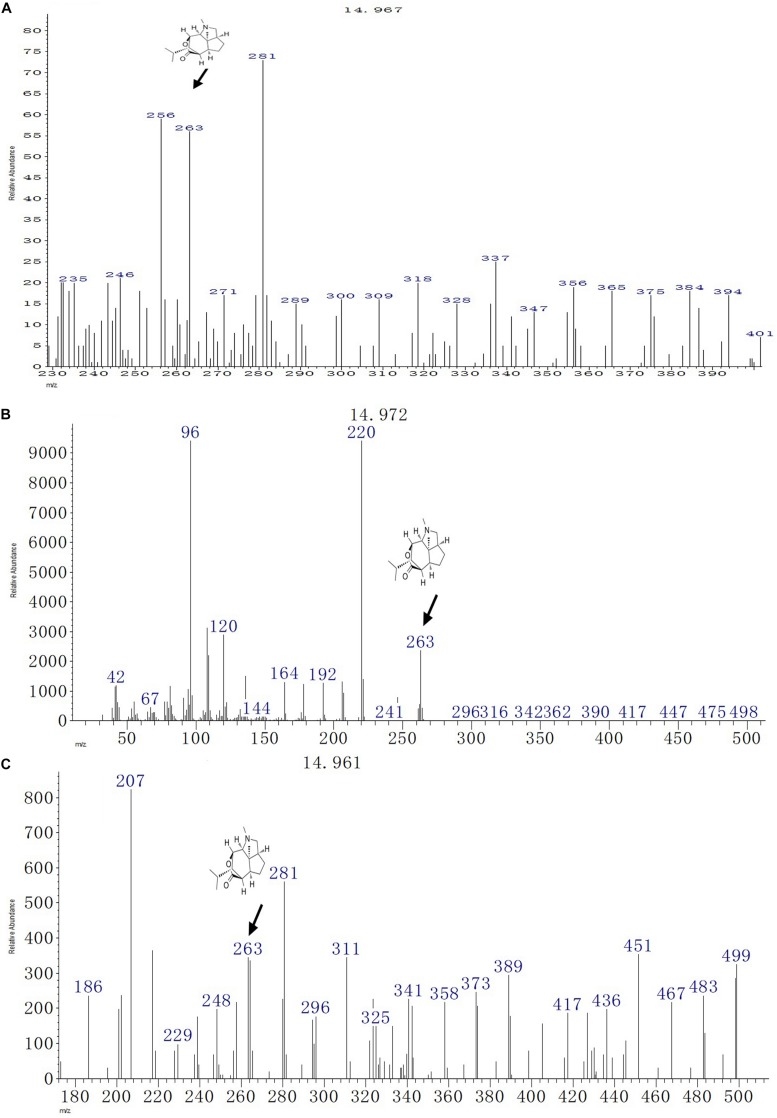
GC-MS chromatographs for the detection of dendrobine (molecular weight: 263). **(A)** *T. longibrachiatum* MD33 dendrobine. **(B)** *Dendrobium nobile* stem dendrobine. **(C)** Chemical reference dendrobine standard.

The GC-MS analysis of the fungal biomass of *T. longibrachiatum* MD33 revealed the presence of dendrobine bioactive volatile compounds. The *T. longibrachiatum* MD33 was found strongly to produce dendrobine as compared with standard chemical reference dendrobine and *D. nobile* stem dendrobine. The mass spectrum of the *T. longibrachiatum* MD33 biomass dendrobine along with *D. nobile* stem dendrobine and chemical reference standard dendrobine showed dendrobine molecular weight at 263 with a peak retention time of 14.96 ± 0.01, respectively (Figure 3 and Table 1). Other compounds produced by *T. longibrachiatum* MD33 were **(1)** 1-Decene, 8- methyl-, **(2)** Benzene, 2-ethyl-1,4- dimethyl-, **(3)** Ethane, hexachloro- (CAS); Hexachloroethane; Egitol; Phenohep; Distopan; Distopin; Falkitol; Distokal; Avlothane; Fasciolin; Mottenhexe; Perchloroethane; Hexachlorethane; Hexachloroethylene; Carbon hexachloride; Ethane hexachloride; 1,1,1,2,2,2-Hexachloro, **(4)** Benzene, 1,2,4,5-tetramethyl-; Durene; Durol; 1,2,4,5-Tetramethylbenzene, **(5)** Benzene, 1,2,3,4-tetramethyl- (CAS); Prehnitol; 1,2,3,4-Tetramethylbenzene; Prehnitene; 1,2,3,4-Tetramethylbenzene (Prehnitene), **(6)** Dodecane, **(7)** Dodecane, 4,6- dimethyl-, **(8)** Benzene, 1,3-bis(1,1-dimethylethyl)-, **(9)** Eicosane, **(10)** Hexane, 2,3,4- trimethyl-, **(11)** 2-Undecene, 4,5- dimethyl-, [R^∗^,S^∗^-(Z) ]-, **(12)** Hexane, 2,3,4- trimethyl-, **(13)** Tetradecane (CAS); n-Tetradecane; Isotetradecane, **(14)** Ethanone, 1,1′-(1,4-phenylene)bis-; Benzene, p-diacetyl-; p-Acetylacetophenone; p-Diacetylbenzene; 1,4-Diacetylbenzene, **(15)** Tridecane, 1- iodo-, **(16)** Pentadecane, **(17)** Phenol, 2,4-bis(1,1-dimethylethyl)-, **(18)** Pyridine-3-carboxamide, oxime, N-(2-trifluoromethylphenyl)-, **(19)** Sulfurous acid, octadecyl 2-propyl ester, **(20)** Cyclohexane, 1,2,4- trimethyl-, **(21)** Hexadecane, **(22)** Heptadecane, **(23)** Octadecane (CAS); n-Octadecane; Octadecan, **(24)** 2-Isopropyl-5-methyl-1-heptanol, **(25)** Eicosane, **(26)** Nonadecane, **(27)** Octadecane (CAS); n-Octadecane; Octadecan, **(28)** Hexadecane, 3-methyl-; 3-Methylhexadecane, **(29)** Heptadecane, **(30)** Hexadecanoic acid (CAS); Palmitic acid; Palmitinic acid; n-Hexadecoic acid; n-Hexadecanoic acid; Pentadecanecarboxylic acid; 1-Pentadecanecarboxylic acid; Prifrac 2960; Coconut oil fatty acids; Cetylic acid; Emersol 140; Emersol 143; Hexadecylic acid; Hyd, **(31)** 1,2-Benzenedicarboxylic acid, ditridecyl ester, **(32)** Eicosane (CAS); n-Eicosane, **(33)** Heneicosane, **(34)** Heneicosane, **(35)** Oleic Acid; 9-Octadecenoic acid (Z)-;.delta.(Sup9)-*cis*-Oleic acid; *cis*-.delta.(Sup9)-Octadecenoic acid; *cis*-Oleic Acid; cis-9-Octadecenoic Acid; Emersol 211; Emersol 220 White Oleic Acid; Emersol 221 Low Titer White Oleic Acid; Oelsauere; Oleine 7503; Pa, **(36)** Oleic Acid; 9-Octadecenoic acid (Z)-;.delta.(Sup9)-*cis*-Oleic acid; *cis*-.delta.(Sup9)-Octadecenoic acid; *cis*-Oleic Acid; *cis*-9-Octadecenoic Acid; Emersol 211; Emersol 220 White Oleic Acid; Emersol 221 Low Titer White Oleic Acid; Oelsauere; Oleine 7503; Pa, **(37)** Thiosulfuric acid (H2S2O3), S-(2-aminoethyl) ester; Thiosulfuric acid, S-(2-aminoethyl) ester; Cysteamine, S-sulfo-; Cysteaminesulfonic acid; S-.beta.-Aminoethylthiosulfuric acid; S-(2-Aminoethyl) hydrogen thiosulfate; 2-Aminoethanethiol hydrogen sulfate, **(38)** Triacontane (CAS); n-Triacontane, **(39)** Tricosane (CAS); n-Tricosane, **(40)** Octadecane (CAS); n-Octadecane; Octadecan, **(41)** Phenol, 2,2′-methylenebis[6-(1,1-dimethylethyl)-4-methyl- (CAS); 2,2′-Methylenebis(4-methyl-6-tert-butylphenol); BKF; AO 1; S 67; CAO 5; CAO 14; CAO-14; AO 2246; A-22-46; NG 2246; A 22-46; MBP 5; 2,2′-METHYLENE-BIS(6-T-BUTYL-P-CRESOL); Anti Ox; Catolin 14, **(42)** Eicosane (CAS); n-Eicosane, **(43)** Nonadecane.

### Detection of Dendrobine by LC-MS/MS Analysis

**FIGURE 4:**
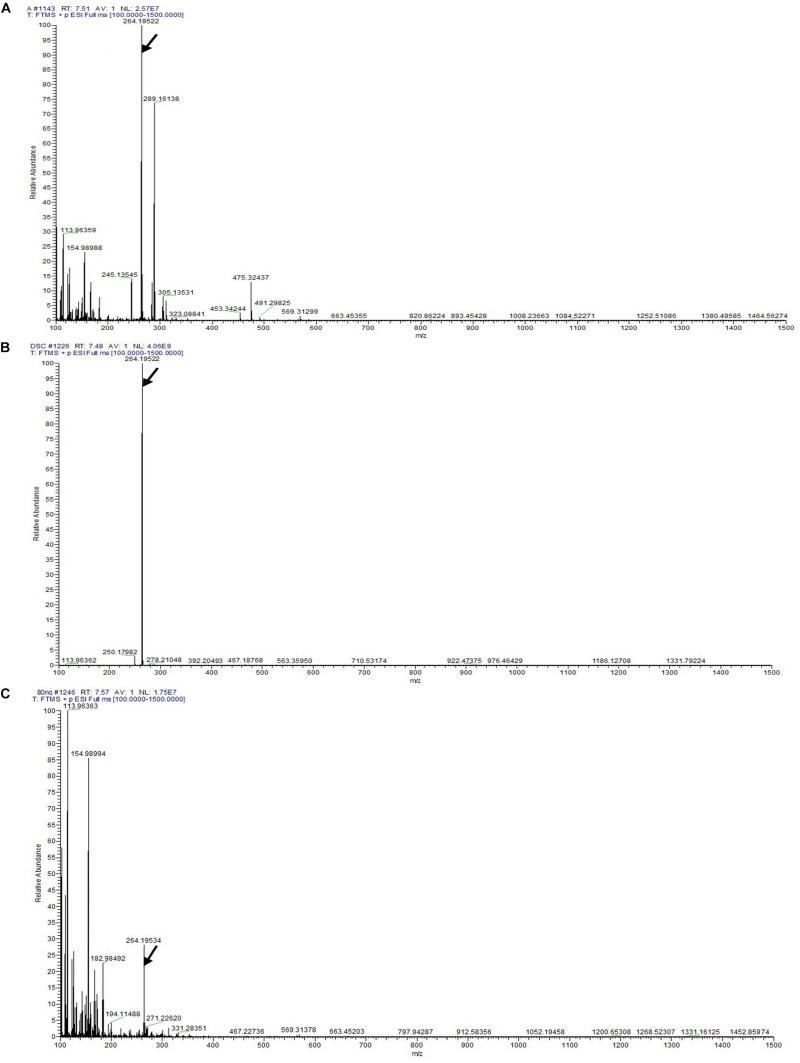
LC-MS/MS chromatographs for the detection of dendrobine (molecular weight: 264.195). **(A)** *T. longibrachiatum* MD33 intracellular dendrobine. **(B)** *Dendrobium nobile* stem dendrobine. **(C)** Chemical reference dendrobine standard.

The LC-MS results revealed that standard chemical reference dendrobine was found at a retention time of 7.57 ± 0.1 and a molecular weight of 264.195. From the *D. nobile* stem dendrobine, it was found that the dendrobine peak was recorded at a retention time of 7.48 ± 0.1 with 264.195 molecular weight, which was nearly the same as the chemical standard dendrobine peak retention time and molecular weight. In case of intracellular dendrobine of *T. longibrachiatum* MD33, the dendrobine peak was recorded at 7.51 ± 0.1 with a dendrobine molecular weight of 264.195. The *T. longibrachiatum* MD33 was found to strongly produce dendrobine as compared with standard chemical reference dendrobine and *D. nobile* stem dendrobine in the LC-MS/MS analysis (Figure 4 and Table 1).

### Toxic Effect of Fungal Metabolite Against Phytopathogenic Bacteria

The *T. longibrachiatum* MD33 strain metabolite showed potent toxicity against all tested bacteria. The *T. longibrachiatum* MD33 exhibited the highest inhibition zone size of 12 ± 0.2 mm against *B. subtilis*, followed by *B. mycoides* and *Staphylococcus* sp. with inhibition zone sizes of 9 ± 0.2 and 8 ± 0.2 mm, respectively, indicating weaker effects. The findings are presented in Table 2. The results showed that *T. longibrachiatum* MD33 possessed potent toxic effects against plant pathogens such as *B. subtilis*, *B. mycoides*, and *Staphylococcus* sp. It was found that the *T. longibrachiatum* MD33 not only produced many bioactive compounds but also showed antibacterial activity of these natural compounds.

**TABLE 2:** Toxicological effect of *Trichoderma longibrachiatum* metabolite against test pathogenic bacteria.

Phytopathogenic bacteria	Test fungi	Zone of inhibition	Zone (in mm)
*Bacillus subtilis*	*Trichoderma longibrachiatum*	+	12 ± 0.2 mm
*Bacillus mycoides*		+	9 ± 0.2 mm
*Staphylococcus* sp.		+	8 ± 0.2 mm

*Values are presented as means ± standard deviation (S.D.); + = Produced inhibition zone; - No zone of inhibition.*

## Discussion

In this study, we aimed to determine the presence of *T. longibrachiatum* MD33 endophytic fungal strain in *D. nobile* stems, to assess the potential of the fungi to produce dendrobine compounds, and to determine the antibacterial effects of bioactive compounds against phytopathogenic bacteria. We found that *T. longibrachiatum* MD33 was involved in the production of a similar bioactive compound of *D. nobile* such as dendrobine. Moreover, dendrobine exhibited strong antibacterial activity against the bacterial strains tested. *D. nobile*, a medicinal plant, may harbor a complex group of endophytic fungi, and previous studies revealed that over 1 million fungi were associated with medicinal plants (Manganyi et al., 2018; Sarsaiya et al., 2020). When these endophytes exist in the internal tissues of medicinal plants, they can form and secrete secondary metabolites that may be of great value, especially in pharmaceutical and agricultural applications (Ghorbanpour et al., 2018). The current study was therefore designed to elucidate the *T. longibrachiatum* MD33 endophyte isolated from *D. nobile* segments and to screen the isolates for dendrobine compound and determine their antibacterial activities against plant pathogens. The fungal endophytes and inner herb tissues create a favorable environment for each other. The versatile synthesis capabilities of fungi are favored to their absorptive and heterotrophic mode of nutrition (Suryanarayanan et al., 2009; Sarsaiya et al., 2019b). This observation is in line with a previous research on endophytic fungi from plant segments (Kosawang et al., 2018).

In the current study, we found that endophytic fungal establishment upsurges in plant segments, because it depends on micro- and macronutrients and other climatic conditions. It was found that the colonization of *Trichoderma* endophytic fungi was higher in stem segments, indicating that the stem is more suitable for the colonization of *Trichoderma* as compared with other endophytic fungi. The outline of isolations and variations in fungal colonization according to plant segments validates that the community of endophytes in *D. nobile* herb is distributed on different segments of the host herb through spore dispersion in the environment. Henceforth, among other aspects, contact time, size of interaction surface, and the amount of natural intros in the plant tissue may impact the quantity of endophytic formations (Nascimento et al., 2015). Unlike bacteria, the identification and organization of fungi still rely mostly on morphological features and the use of light microscope relics to be dynamic in fungiform investigations. However, such identification techniques may be problematic and time-consuming as they may need specific proficiency to obtain consistent evidence (Ziaee et al., 2016). In the current research, both macroscopic features and microscopic practices were used to identify fungal isolate. The present study suggests that ITS sequencing provides an adequate resolution to identify endophytic fungi at least to the species level. The results showed that the isolated endophytic fungus from the stem segment of wild *D. nobile* was *T. longibrachiatum* MD33. Similarly, *Trichoderma viride* strain was reported from different medicinal plant segments previously (Nalini et al., 2014; Xiang et al., 2016).

Fungal endophytes from *D. nobile* are still a weakly investigated set of microorganisms, and their complex biological functions remain poorly elucidated (Zheng et al., 2017). *D. nobile* is a medicinal plant used widely in China. To date, there are no reports on dendrobine and related compound-producing endophytic fungi isolated from *D. nobile*; however, studies have focused on dendrobine produced by the herb (Li et al., 2017; Meng et al., 2017). *D. nobile* has attracted increasing attention in medical and chemical fields because of its active ingredients. However, the production of these ingredients in the plants is very low. A few endophytic fungi have the ability to produce substances similar to the host herb. Another research revealed that the taxol bioactive compound had been detected as an alternative source of natural compound (earlier obtained from Taxus plant) from endophytic fungal species, which has shown antimicrobial and anti-cancer activity (Somjaipeng et al., 2015). This attracted interest in the potential of endophytes isolated from *D. nobile* to produce a high value product such as dendrobine. Owing to the increasing global demand for anti-cancer drugs, the global market for dendrobine is increasing rapidly and is expected to cross the outer limit of other drugs. Thus, the demand for dendrobine will continue to increase (Li et al., 2017; Sarsaiya et al., 2019c). However, dendrobine is an expensive drug and, therefore, not easily accessible to many people worldwide. To our knowledge, no attempt has been made to isolate endophytes from *D. nobile* for possessing the ability to produce dendrobine in China and other countries. Through HPLC analysis, it was observed that *T. longibrachiatum* MD33 showed a peak retention time (5.28 ± 0.2 min) similar to the host plant (*D. nobile*) and standard chemical reference dendrobine. This finding suggested the possibility of finding alternative sources of dendrobine through fungal endophyte (*T. longibrachiatum* MD33). The GC-MS of the *T. longibrachiatum* MD33 biomass dendrobine along with *D. nobile* stem dendrobine and standard chemical reference dendrobine showed similar dendrobine (263 molecular weight and 14.96 ± 0.01 retention time). In the case of LC-MS/MS, the *T. longibrachiatum* MD33 was found strongly to produce dendrobine (264.195 molecular weight and 7.51 ± 0.1 retention time) as compared with standard chemical reference dendrobine and *D. nobile* stem dendrobine. Previous research on the metabolite profile of *D. nobile* has revealed that dendrobine, an alkaloid found in *D. nobile*, is a hydrophobic weak base, related to the picrotoxin family of natural products, and has a molecular weight of 263 in GC-MS (Yamamura and Hirata, 1964) and 264.195 in LC-MS, UHPLC system (Wang et al., 2016), with a chemical structure of C_16_H_25_NO_2_. The findings of the present study will be an important research breakthrough to fulfill the market demands of dendrobine production in the future.

It is well known that endophytic fungi are considered “biofactories” of bioactive products for the development of natural products besides active biological mediators (ABMs) compared to phytopathogens, because they show beneficial functions on host herbs. In the present study, the biocontrol potential of cultivable endophytic fungi harbored in *D. nobile* was described. Furthermore, *Trichoderma* species are currently utilized as growth promoters or bio-fertilizers in the agricultural industry (Manganyi et al., 2018). Our present study showed that *T. longibrachiatum* MD33 strain was effective for growth mitigation of phytopathogenic bacteria found in the rhizospheric wastewater-contaminated soil. The *T. longibrachiatum* MD33 was effective for inhibiting the growth of most of the pathogenic bacteria tested (*B. subtilis*, *B. mycoides*, and *Staphylococcus* sp.). These findings are in accordance with the results of Nwakanma et al. (2016). The previous findings revealed that fungal endophytes belonging to *Aspergillus* (Mousavi et al., 2016) and *Penicillium* (Chowdhary et al., 2016) genus have shown antibacterial activity. Our study has also shown that *T. longibrachiatum* MD33 also contained compounds that were active against plant bacterial pathogens and rhizosphere soil-contaminated (wastewater) pathogenic microorganisms. We suggest that fungal endophytes can play important functions to support the survival of host herbs against development of phytobacterial diseases because the contaminated wastewater has favored the existence of pathogenic bacteria in the rhizosphere regions. The *T. longibrachiatum* MD33 metabolite can be used for the cleanup of pathogenic bacteria for plants as well as from the wastewater.

Finally, dendrobine, a major component of *D. nobile*, but now from *T. longibrachiatum* MD33 (as an alternative source of dendrobine), has caught the attention of researchers worldwide for its wide applications in the pharmaceutical and medical field. More importantly, the results provide valuable information on the full practical application of HPLC, GC-MS, and LC-MS/MS for the accurate detection of dendrobine as compared with *D. nobile* stem and standard chemical reference dendrobine (Figures 2–4). Dendrobine was firstly extracted in 1932 (from *D. nobile* Lindl) through detection from the Chinese herb *D. nobile*. The chemical structural formula of dendrobine was C_16_H_25_NO_2_, molecular weight 263, melting at 134–136°C, soluble in chloroform, and insoluble in ethanol and water (Yamamura and Hirata, 1964). However, there is still no report on the alternative production of dendrobine from fungal sources. In this study, we recognized a simple, accurate, and rapid method for the chloroform extraction and detection of dendrobine in *T. longibrachiatum* MD33 using HPLC, GC-MS, and LC-MS/MS. Chloroform is an active solvent for natural bioactive compounds in their base form and thus fungal material is regularly extracted with chloroform for bioactive compound investigation. Dendrobine bioactive compound is strictly hydrophobic. Chloroform extraction is used for extraction of dendrobine bioactive compound. Furthermore, HPLC was used for the preliminary detection of dendrobine, but for strong chemical and structural confirmation, the GC-MS (*T. longibrachiatum* MD33 dendrobine molecular weight 263) and LC-MS/MS (*T. longibrachiatum* MD33 dendrobine molecular weight 264.195) techniques gave clear evidence of *T. longibrachiatum* MD33 dendrobine, which was never seen in any science research database. In the future, the pure form of *T. longibrachiatum* MD33 dendrobine will be used for the determination of dendrobine function and applications for pharmacokinetic investigation, which will give more insights into the function and applicability of the *T. longibrachiatum* MD33 dendrobine.

In summary, the dendrobine produced by *T. longibrachiatum* MD33 endophytic fungi was reported for the first time. The characterization of *T. longibrachiatum* MD33 biomass using highly accurate HPLC, GC-MS, and LC-MS/MS analysis showed that the similar peak retention time and molecular weight of dendrobine were observed as compared with standard chemical reference dendrobine and *D. nobile* stem dendrobine, indicating the strong evidence of the presence of dendrobine. Furthermore, *T. longibrachiatum* MD33 endophytic fungi metabolite showed antibacterial effects against phytopathogenic bacteria. In conclusion, this study can lay the foundation for therapeutic application of an alternative source of dendrobine from *T. longibrachiatum* MD33 endophytic fungi in medical and biotechnology fields.

## Data Availability Statement

The datasets generated for this study are available on request to the corresponding author.

## Author Contributions

SS, JC, and JS conceived and designed the experiments. SS, AJ, and XF performed the experiments. SS and JC analyzed the data. QJ, FS, QZ, and QX contributed reagents, materials, and analysis tools. SS wrote and edited the manuscript.

## Conflict of Interest

The authors declare that the research was conducted in the absence of any commercial or financial relationships that could be construed as a potential conflict of interest.

## Footnotes

1 http://blast.ncbi.nlm.nih.gov/Blast.cgi

## Abbreviations

ANOVA: analysis of variance
BLAST: Basic Local Alignment Search Tool
GC-MS: gas chromatography–mass spectrometry
HPLC: high-performance liquid chromatography
ITS: internal transcribed spacer
LC-MS/MS: liquid chromatography with tandem mass spectrometry
NCBI: National Center for Biotechnology Information
PCR: polymerase chain reaction
PDA: potato dextrose agar
PDM: potato dextrose medium
S.D.: standard deviation.
